# Pathology of asthma

**DOI:** 10.3389/fmicb.2013.00263

**Published:** 2013-09-10

**Authors:** Makoto Kudo, Yoshiaki Ishigatsubo, Ichiro Aoki

**Affiliations:** ^1^Department of Clinical Immunology and Internal medicine, Graduate School of Medicine, Yokohama City UniversityYokohama, Japan; ^2^Department of Pathology, Graduate School of Medicine, Yokohama City UniversityYokohama, Japan

**Keywords:** asthma, remodeling, epithelial to mesenchymal transition, Th2 cells, cytokines, Th17 cells, Th9 cell

## Abstract

Asthma is a serious health and socioeconomic issue all over the world, affecting more than 300 million individuals. The disease is considered as an inflammatory disease in the airway, leading to airway hyperresponsiveness, obstruction, mucus hyper-production and airway wall remodeling. The presence of airway inflammation in asthmatic patients has been found in the nineteenth century. As the information in patients with asthma increase, paradigm change in immunology and molecular biology have resulted in an extensive evaluation of inflammatory cells and mediators involved in the pathophysiology of asthma. Moreover, it is recognized that airway remodeling into detail, characterized by thickening of the airway wall, can be profound consequences on the mechanics of airway narrowing and contribute to the chronic progression of the disease. Epithelial to mesenchymal transition plays an important role in airway remodeling. These epithelial and mesenchymal cells cause persistence of the inflammatory infiltration and induce histological changes in the airway wall, increasing thickness of the basement membrane, collagen deposition and smooth muscle hypertrophy and hyperplasia. Resulting of airway inflammation, airway remodeling leads to the airway wall thickening and induces increased airway smooth muscle mass, which generate asthmatic symptoms. Asthma is classically recognized as the typical Th2 disease, with increased IgE levels and eosinophilic inflammation in the airway. Emerging Th2 cytokines modulates the airway inflammation, which induces airway remodeling. Biological agents, which have specific molecular targets for these Th2 cytokines, are available and clinical trials for asthma are ongoing. However, the relatively simple paradigm has been doubted because of the realization that strategies designed to suppress Th2 function are not effective enough for all patients in the clinical trials. In the future, it is required to understand more details for phenotypes of asthma.

## INTRODUCTION

Asthma is characterized by the action of airway leading to reversible airflow obstruction in association with airway hyperresponsiveness (AHR) and airway inflammation (Holgate, 2012). The disease is affecting more than 300 million persons all over the world, with approximately 250,000 annual deaths (Bousquet et al., 2007). In the last couple of decades, as the inhaled corticosteroid has become the major treatment agent for asthma, the mortality of asthma has decreased (Wijesinghe et al., 2009). Meanwhile, allergic diseases, such as asthma, have markedly increased in the past half centuries associated with urbanization (Alfvén et al., 2006). Children have the greatest percentage of asthma compared with other generation groups (Centers for Disease Control and Prevention, 2011). Then, it is expected that the number of the patients will increase by more than 100 million by 2025 (Masoli et al., 2004).

Generally, most asthma starts from childhood in relation to sensitization to common inhaled allergens, such as house dust mites, cockroaches, animal dander, fungi, and pollens. These inhaled allergens stimulate T helper type 2 (Th2) cell proliferation, subsequently Th2 cytokines, interleukin (IL)-4, IL-5 and IL-13 production and release. Many basic and clinical studies suggested that airway inflammation was a central key to the disease pathophysiology. The existence of chronic airway inflammation in asthma has been recognized for over a century. The inflammation is induced by the release of potent chemical mediators from inflammatory cells. Resulted of chronic airway inflammation, airway remodeling, characterized by thickening of all compartments of the airway wall, is occurred and may have profound consequences on the mechanics of airway narrowing in asthma and contribute to the chronicity and progression of the disease.

As allergic sensitization, allergen can be taken up by dendritic cells (DCs), which process antigenic molecules and present them to naїve T helper cells. Consequently the activation of allergen-specific Th2 cells is occurred, the cells play an important role in developing the asthma. Nowadays, it is known that Th17 cells and Th9 cells also modulate the disease. Th17 cells produce IL-17A, IL-17F, and IL-22. These cytokines induce airway inflammation and IL-17A enhance smooth muscle contractility.

Allergic diseases are caused by inappropriate immunological responses to allergens without pathogenesis driven by a Th2-mediated immune response. The hygiene hypothesis has been used to explain the increase in allergic diseases since industrialization and urbanization, and the higher incidence of allergic diseases in more developed countries. The hypothesis has now expanded to include exposure to symbiotic bacteria and parasites as important modulators of immune system development, along with infectious agents (Grammatikos, 2008). Recently, asthma has not been recognized as a simple Th2 disease, which is characterized by IgE elevation and relatively eosinophilia. Th17 and Th9 cell subtype are known to contribute the inflammation or enhancing smooth muscle contraction or stimulating mast cells.

## HISTOPATHOLOGY OF ASTHMATIC AIRWAY

Asthma is considered in terms of its hallmarks of reversible airflow obstruction, non-specific bronchial hyperreactivity and chronic airway inflammation (American Thoracic Society, 1987). Osler (1892) mentioned in the classic textbook, the inflammatory process, affecting the conducting airways with relative sparing of the lung parenchyma. Huber and Koesser (1922) provided a comprehensive perspective of the histopathological features of asthma. That is, the lungs are usually hyperinflated as a consequence of extensive mucous plugging in segmental, subsegmental bronchus and peripheral airways, but the lung parenchyma in general, remains relatively intact in subjects who die in exacerbation, so-called *status asthmatics*. The composition of mucous includes cellular debris from necrotic airway epithelial cells, an inflammatory cells including lymphocytes, eosinophils, and neutrophils, plasma protein exudate, and mucin that is produced by goblet cells (Unger, 1945; Bullen, 1952; Dunnill, 1960; Messer et al., 1960). The airway epithelium typically shows sloughing of ciliated columnar cells, with goblet cell and squamous cell metaplasia as a sign of airway epithelial repair. There is increased thickness of the subepithelial basement membrane, however, some studies have established that the true basal lamina is of normal thickness, and the apparent increase in thickness is related to accumulation of other extracellular matrix components beneath the basal lamina (Roche et al., 1989). The asthmatic airway showed a thickness with inflammatory cell infiltration consisting of an admixture of T lymphocytes and eosinophils, mast cells (Carroll et al., 1997; Hamid et al., 1997). Interestingly, prominent neutrophil infiltrates have been reported to be a specific feature of the clinical entity of sudden onset fatal asthma (Sur et al., 1993).

Nowadays investigators can easily obtain lung tissue and bronchoalveolar lavage (BAL) specimens from the patients with asthma (Salvato, 1968; Djukanovic et al., 1991). Results of studies of BAL (Robinson et al., 1992) and lung tissue specimens (Minshall et al., 1998) have strongly implicated a role for cytokines produced by the Th2 subset of CD4+ T cells in the pathogenesis of asthma. For example, IL-13 plays an important role in regulating the airway inflammation in asthma (Wills-Karp et al., 1998; Zhu et al., 1999).

In recent years, there has been increasing interest in the mechanism of airway wall remodeling in asthma, owing to the increasing realization that airway inflammation alone is not enough to explain the chronicity or progression of asthma (Holgate et al., 1999). The nature of airway remodeling may be considered in terms of extracellular matrix deposition. It is postulated that the injured airway epithelium acts as a continuous stimulus for airway remodeling (Holgate et al., 1999), and this is supported by results of recent cell culture experiments examining interactions of bronchial epithelial cells with myofibroblasts in response to injurious stimuli (Zhang et al., 1999). The remodeling is predicted to have little effect on baseline respiratory mechanics, the physiological effects of extracellular matrix accumulation are predicted to result in an exaggerated degree of narrowing for a given amount of airway smooth muscle (ASM) contraction.

Airway wall thickening is greater in the asthmatic patients than normal subjects, and severe patients have greater (Awadh et al., 1998). This thickness is due to an increase in ASM mass and mucous glands (Johns et al., 2000). The airflow limitation is also compounded by the presence of increased mucous secretion and inflammatory exudate (Chiappara et al., 2001). Thus, the results from many studies have supported that airway remodeling related to airway inflammation. Surprisingly, physical force generated by ASM in bronchoconstriction without additional inflammation induces airway remodeling in patients with asthma (Grainge et al., 2011). Despite these recent advances, further work is necessary to establish a causal relationship between airway remodeling and the severity of asthma (Bento and Hershenson, 1998).

## AIRWAY EPITHELIUM

The structural changes in the asthmatic airway result from interdependent inflammatory and remodeling processes (Chiappara et al., 2001). In the processes, inflammation occurs common features, vascular congestion, exudaution, and inflammatory cell recruitment to the interstitial tissue. Furthermore mucus secretion and desquamation of epithelial cells are increased. The chronic inflammatory changes develop epithelium-mesenchymal interactions (Holgate et al., 2000). The number of myofibroblasts, which deposit collagens, increases in the understructure of epithelium, the proximity of the smooth muscle layer and the lamina reticularis in the patients. Subepithelial collagens cause thickening and increasing density of the basement membrane.

The airway inflammation gives damage to the epithelium and damaged epithelial cells will be repaired in the injury-repair cycle. Some studies showed that epithelial cells of untreated asthmatic patients had low level expression of proliferating markers, despite extensive damage, revealing a potential failure in the epithelial injury-repair cycle in response to local inflammation and inhaled agents (Bousquet et al., 2000). Injury to the epithelium results in a localized and persistent increase in epidermal growth factor (EGF) receptor, a mechanism that may cause the epithelium to be locked in a repair phenotype (Puddicombe et al., 2000). Epithelial cells which are in repair phase produced some profibrotic mediators, including transforming growth factor-β (TGF-β), fibroblast growth factor and endothelin, which regulate fibroblast and myofibroblast to release collagen, elastic fiber, proteoglycan, and glycoprotein and these substances induce airway wall thickening (Holgate et al., 2000). Myofibroblast is a rich source of collagen types I, II, and V, fibronectin and tenascin that also accumulate in the airway wall and induce thickening lamina reticularis (Roche et al., 1989; Brewster et al., 1990). This process may contribute phenomena by augmentation of airway narrowing because the inner airway wall volume increases.

Eosinophils seem to contribute to airway remodeling in several ways, including through release of eosinophil-derived TGF-β, cationic proteins, and cytokines, as well as through interactions with mast cell and epithelial cells. Many of these factors can directly activate epithelium and mesenchymal cells, deeply related to the development of airway remodeling (Kariyawasam and Robinson, 2007; Aceves and Broide, 2008; Venge, 2010). Eosinophil-derived cytokines are in the modulation of Th2 responses that trigger macrophage production of TGF-β1, which serves as a stimulus for extracellular matrix production (Fanta et al., 1999; Holgate, 2001). TGF-β1 induced epithelial to mesenchymal transition (EMT) in alveolar epithelial cells and could contribute to enhance fibrosis in idiopathic lung fibrosis (Wilson and Wynn, 2009). TGF-β1 might also contribute to enhance airway remodeling through EMT. Indeed, anti-TGF-β1 treatment inhibits EMT in airway epithelial cells (Yasukawa et al., 2013).

Airway epithelium is a barrier in the frontline against stimuli from the environment, but in asthmatic epithelium is defective in barrier function with incomplete formation of tight junctions, that prevent allergen from penetrating into the airway tissue (Xiao et al., 2011). The defect would induce that a proportion of the asthma-related had biological properties to infiltrate the epithelial barrier and trigger a danger signal to DCs. Components of house dust mite, cockroach, animal, and fungal can disrupt epithelial tight junctions and activate protease-activated receptors (Jacquet, 2011). The defective epithelial barrier function has also been described in the pathophysiology of other allergic disease. Therefore, healthy barrier function is important to avoid sensitization and development in allergic disease.

## AIRWAY SMOOTH MUSCLE

Abnormalities of asthmatic ASM structure and morphology have been described by Huber and Koesser (1922) in the first quarter of twentieth century when they reported that smooth muscle from the patients who died by acute exacerbation was increase much greater than in those who died from another disease. Airflow limitation mainly due to reversible smooth muscle contraction is a most important symptom of the disease. Therefore, ASM plays a material role in asthma. Abnormal accumulation of smooth muscle cells is another mechanism of airway remodeling. Some *in vivo* animal studies confirmed that prolonged allergen exposure increase smooth muscle thickness in the airway (Salmon et al., 1999). It is still unknown whether the phenomenon is occurred by fundamental changes in the phenotype of the smooth muscle cells, is caused by structural or mechanical changes in the non-contractile elements of the airway wall. There are two different ways by which cyclic generation of length and force could influence ASM contracting and airway narrowing. The processes, which are myosin binding and plasticity, have different biochemical and physical mechanisms and consequences. They have the potential to interact and to have a fundamental effect on the contractual capacity of smooth muscle and its potential to cause excessive airway narrowing (King et al., 1999).

**FIGURE 1:**
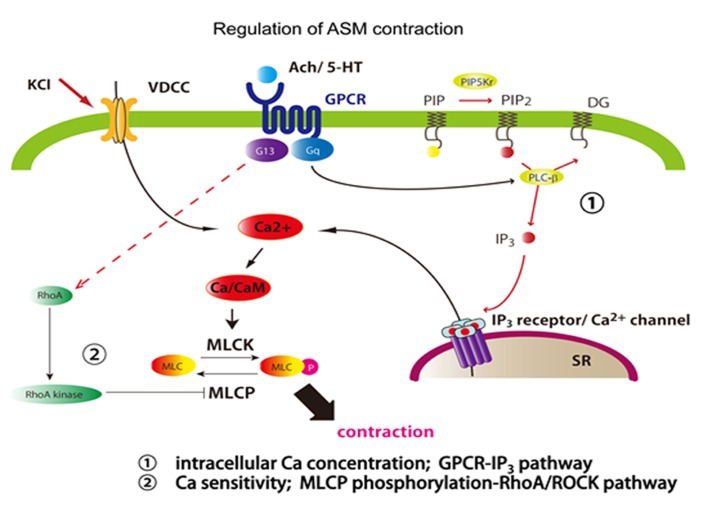
**Regulation of ASM contractility**. ASM contraction is induced by calcium, regulated two different pathways. First, ASM is evoked by intracellular calcium influx from SR depending on GPCR stimulation or from the extracellular environment through voltage-dependent calcium channel. Second, smooth muscle can be induced calcium sensitivity by RhoA/Rho kinase pathway. RhoA activates Rho-kinase which phosphorylates MLCP. pMLC phosphatase fails to dephosphorylate MLC. KCl, potassium chloride; Ach, acetylcholine; 5-HT, 5-hydroxytryptamine (serotonin); PIP, phosphatidylinositol 4-phosphate; PIP2, phosphatidylinositol 4,5-bisphosphate; PIP5K, 1-phosphatidylinositol-4-phosphate 5-kinase; DG, diacylglycerol; IP3, inositol 1,4,5-trisphosphate.

Like other muscles, ASM is also provoked to contract with intracellular calcium ions (Ca^2+^), which comes from the extracellular environment through voltage-dependent calcium channel or from the sarcoplasmic reticulum stores (**Figure 1**). The source of Ca^2+^ surge in ASM is mainly from intracellular sarcoplasmic reticulum stores rather than from the extracellular Ca^2+^ seen in cardiac, skeletal, and vascular muscle cells. Ligands to G-ptotein coupled receptor (GPCR), such as acetylcholine and methacholine, induce the activation of phospholipase C (PLC), which in turn leads to the formation of the inositol triphosphate (IP_3_; Chen et al., 2012). Then, IP_3_ occurs to release Ca^2+^ from sarcoplasmic reticulum (SR) stores, then Ca^2+^ forms a calcium-calmodulin comlex, activates MLC kinase (MLCK) which phosphorylates regulatory MLCs (rMLCs) forming phosphorylated-MLC (p-MLC; Berridge, 2009). Finally, this mechanism occurs to the activation of actin and myosin crossbridges resulting in shortening and contraction (Gunst and Tang, 2000).

And the contraction is also regulated by calcium sensitivity of myosin light chain (MLC; Kudo et al., 2012). The p-MLC is regulated by MLC phosphatase (MLCP) which converts p-MLC back to inactive MLC. MLCP is negatively controlled by Ras homolog gene family, member A (RhoA) and its target Rho Kinase such as Rho-associated, coiled-coil containing protein kinase (ROCK) which phosphorylates myosin phosphatase target subunit 1 (MYPT-1). Upregulation of the RhoA/Rho kinase signaling pathway inducing to inhibition of MLCP would result in increased levels of p-MLC and subsequently increased ASM contraction force. Increased levels of RhoA protein and mRNA were found in airway hyperresponsive animal models and this is probably medicated through inflammatory cytokines, such as IL-13 and IL-17A that themselves directly enhance the contractility of ASM (Chiba et al., 2009; Kudo et al., 2012). For IL-17A, sensitized mouse conditional lacking integrin αvβ8 on DCs shows attenuated reactivity against IL-17A-induce antigen challenge. This is induced by that IL-17A itself enhances the contractile force of ASM, through RhoA/Rho kinase signaling change.

Airway smooth muscle cells also contribute to the inflammatory mechanisms and airway remodeling of asthma. The proactivating signals, including viruses and immunoglobulin E could convert ASM cells into a proliferative and secretory cell in asthma. Naureckas et al. (1999) demonstrated the presence of smooth muscle mitogens in the BAL fluids from asthmatic individuals who underwent allergen challenge. Smooth muscle proliferation is also caused by the production of matrix metalloproteinase (MMP)-2, which has been demonstrated to be an important autocrine factor that is required for proliferation (Johnson and Knox, 1999). Production of MMP-2 from smooth muscle cells suggests that ASM contributes to the extracellular matrix turnover and airway remodeling. These cells may also participate in chronic airway inflammation by interacting with both Th1- and Th2-derived cytokines to modulate chemoattractant activity for eosinophils, activated T lymphocytes, and monocytes/macrophages (Teran et al., 1999).

In addition, recent studies demonstrated that eosinophils can also contribute to airway remodeling during an asthma by enhancing ASM cell proliferation. Halwani et al. (2013) verified that preventing eosinophil contact with ASM cells using specific antibodies or blocking cysteinyl leukotrienes derived from eosinophils was associated with inhibition of ASM proliferation. Moreover, ASM-synthesized cytokines seem to direct the eosinophil differentiation and maturation from progenitor cells, which can promote perpetuation of eosinophilic inflammation and consequently the tissue remodeling in asthma (Fanat et al., 2009). It was also reported that TGF-β alone induces only weak mitogenic effect on ASM cells, however, it synergistically stimulates ASM proliferation with methacholine which is agonist for the muscarinic receptor (Oenema et al., 2013). These smooth muscle cell proliferations related to airway remodeling can be the target to treat asthma.

## EPITHELIAL TO MESENCHYMAL TRANSITION ON ASTHMA

As airway remodeling on asthma attracts investigators interested in airway remodeling on asthma, EMTs are recognized to be more important in asthma than before. EMTs are biological processes that epithelial cells lose their polarity and cell adhesion resulted in fragility of tight junction and gain migratory and invasive properties to change their cell formation to mesenchymal cells (Kalluri and Neilson, 2003). It is essential for processes including mesoderm formation and neural tube formation in the development and recently has also been reported to involve in wound healing, in organ fibrosis and in cancer metastasis. First, EMTs were found in the embryogenesis. Epithelial cells are different from mesenchymal cells in their phenotype. Epithelial cells connect each other, forming tight junction. These cells have polarity in cytoskeleton and bound to basal lamina. For mesenchymal cells, the polarity is lost and shaped in spindle. Lately, EMTs are divided into three subtypes, developmental (Type I), fibrosis, tissue regeneration and wound healing (Type II), and cancer progression and metastasis (Type III; Kalluri and Weinberg, 2009).

Type II EMT involves in wound healing, resulted that it contributes airway remodeling in asthma after airway epithelial injury induced by inflammation. Type II EMT indicates that epithelial tissue can be expressed plasticity (Thiery and Sleeman, 2006). It is initiated by extracellular signals, such as connection with extracellular matrix; collagen or hyaluronic acids and by growth factors; TGF-β and EGF. Among those signals, TGF-β is established how it plays important role in airway remodeling and EMT (Phipps et al., 2004; Boxall et al., 2006; Hackett et al., 2009). TGF-β induces the expression of α-smooth muscle actin and vimentin and the downregulation of E-cadherin expression, inducing the dissolution of polarity of the epithelial cell and intercellular adhesion. The such physiological effects of TGF-β signaling in the system have been shown to depend on microenvironment. Bone morphogenesis protein (BMP)-7 fails to attenuate TGF-β-induced EMT, however, one of the family member BMP-4 plays the role of EMT in the airway (Molloy et al., 2008; Hackett et al., 2009). This TGF-β-induced attenuation of intercellular adhesion and wound repair in EMT can be enhanced by the proinflammatory cytokines tumor necrosis factor (TNF)-α (Camara and Jarai, 2010). Furthermore, it was showed that house dust mite, through EGF receptor enhanced TGF-β-induce downregulation of E-cadherin in the bronchial epithelial cells (Heijink et al., 2010). And house dust mite and TGF-β synergistically induced expression of mesenchymal markers vimentin and fibronectin. In chronic house dust mite-exposure model, the airway epithelial cells were shown to elevate TGF-β expression and nuclear phosphorylated Smad3. And in these cells, the tight-junction protein was dissolved, occluding and expressed α-smooth muscle actin and collagen (Johnson et al., 2011). Inhaled allergens might modify EMT, cooperating with cytokines which also promote asthma.

## MAST CELLS AND EOSINOPHILS

Mact cells can induce the activation of mesenchymal cells (Holgate, 2000). The serine protease, tryptase which is released from degranulating mast cells is a potent stimulant of fibroblast and smooth muscle cell proliferation, and is capable of stimulating synthesis of type I collagen by human fibroblasts. A major mechanism involved in the regulation of fibroblast proliferation appears to be cleavage and activation of protease activated receptor-2 on fibroblasts (Akers et al., 2000). Mast cells may also influence the development of airway remodeling in asthma by releasing large amounts of plasminogen activator inhibitor type1. Moreover, Sugimoto et al. (2012) have shown that other mast cell proteases regulate airway hyperreactivity. Mice lacking αvβ6 integrin are protected from exaggerated airway narrowing. Mast cell proteases are differentially expressed, in mouse mast cell protease 1 (mMCP-1) induced by allergen challenge in wild-type (WT) mice and mMCP-4 increased at baseline in β6-deficient mice. MCPs from intraepithelial mast cell and their proteolytic substrates could be regulate airway hyperreactivity.

Eosinophils are circulating granulocytes and at relatively low levels in the bloodstream, upto 3% of white blood cells. These are the major cell types that can be recruited to sites of inflammatory responses (Huang et al., 2009; Isobe et al., 2012; Uhm et al., 2012). The function of eosinophils in asthma is related to their release of toxic granule proteins, reactive oxygen species (ROS), cytokines, and lipid mediators (Liu et al., 2006). The recruit of eosinophils into the epithelium and eosinophilic inflammation is involved in the pathogenesis of asthma. The proinflammatory mediators derived by eosinophil are major contributors to inflammation in asthma, including airway epithelial cell damage and desquamation, airway dysfunction of cholinergic nerve receptors, AHR, mucus hypersecretion, and airway remodeling, characterized by fibrosis and collagen deposition (Kay, 2005; Watt et al., 2005; Kanda et al., 2009; Walsh, 2010). Eosinophils are likely to contribute to airway remodeling with release of eosinophil-derived mediators such as TGF-β, secretion of cationic proteins, and cytokines, as well as having interactions with mast cell and epithelial cells. Those factors can directly activate epithelium and mesenchymal cells (Venge, 2010). Moreover, recent data demonstrated that eosinophils can also contribute to airway remodeling with ASM cell proliferation.

## EXTRACELLULAR MATRIX

The airways of asthmatic patients showed excess accumulation of extracellular matrix components, particularly collagen, in the subepithelial connective tissue and adventitia of the airway wall (Kuwano et al., 1993; Gillis and Lutchen, 1999). The cellular interactions in mast cells and fibroblasts through protease activated receptor-2 may contribute an abnormal mesenchymal cell proliferation, and may account for the increased number of fibroblasts and myofibroblasts that are found in the airways of asthmatic subjects. Fibroblasts retain the capacity for growth and regeneration, and may evolve into various cell types, including smooth muscle cells that subsequently become myofibroblasts. Myofibroblasts can contribute to tissue remodeling by releasing extracellular matrix components such as elastin, fibronectin and laminin (Vignola et al., 2000). It was seen that the numbers of myofibroblasts in the airway of asthmatic subjects increased and their number appeared to correlate with the size of the basement reticular membrane (Holgate et al., 2000). Smooth muscle cells also have the potential to alter the composition of the extracellular matrix environment. The reticular basement membrane thickening is a characteristic typical feature of the asthmatic airways. It appears to consist of a plexiform deposition of immunoglobulins, collagen types I and III, tenascin and fibronectin (Jeffery et al., 2000), but not of laminin.

Remodeling processes of the extracellular matrix are less known than the thickening of the lamina reticularis. Most asthmatic subjects present with an abnormal superficial elastic fiber network, with fragmented fibers (Bousquet et al., 2000). In the deeper layer of elastic fibers is also abnormal, the fibers often being often patchy, tangled, and thickened. Some studies using transmission electron microscopy have shown that an elastolytic process occurs in asthmatic patients, and in some patients disruption of fibers has been observed. In the case of fatal asthma, fragmentation of elastic fiber has also been found in central airways, and was associated with marked elastolysis (Mauad et al., 1999). These bundles are seen to be hypertrophied as a result of an increased amount of collagen and myofibroblast matrix deposition occurring during exaggerated elastic fiber deposition (Carroll et al., 1997). Loss of lung elastic recoil force has been shown in adults with persistent asthma and irreversible expiratory airflow obstruction. Persistent asthmatic patients have severe abnormal flow-volume curves in expiration at both high and low lung volumes, and hyperinflation can be seen by residual volume, at forced residual capacity and total lung capacity (Gelb and Zamel, 2000). The increased elastolysis is part of a more complex process that regulates the size of a submucosal network formed by elastic fibers dispersed in a collagen and myofibroblast matrix (Chiappara et al., 2001). These features induce changes in airway, as demonstrated by airway compliance, particularly in those patients who are suffering from asthma for long period, supporting the concept that chronic inflammation and remodeling of the airway wall may result in stiffer dynamic elastic properties of the asthmatic airway (Brackel et al., 2000). Furthermore, disruption of elastic fibers may contribute to a reduction in the preload and afterload for smooth muscle contraction. Though it is difficult to associate aspects of remodeling with disease severity or degree of airways obstruction and hyperresponsiveness (Mauad et al., 2007), some investigators indicated that smooth muscle remodeling is related to the severity of asthma (James et al., 2009). It has shown that the clinical expression of asthma (Brightling et al., 2002), AHR (Siddiqui et al., 2008) and impaired airway relaxation (Slats et al., 2007) are associated with mast cell counts in the ASM layer in asthma. The deposition of extracellular matrix inside and outside the smooth muscle layer in asthma also seems to be related to its clinical severity and is altered as compared to healthy controls (Araujo et al., 2008; Klagas et al., 2009). Yick et al. (2012) have shown that extracellular matrix in ASM was related to the dynamics of airway function in asthma.

## IMMUNE RESPONSE

### ALLERGIC SENSITIZATION

Regarding to the immune system against allergy, it seems that hygiene hypothesis would provide the reason why the number of the patients with asthma is increasing, in relation with urbanization. The hypothesis is that the Th1 cells polarized response is not induced early in life leaving the body more susceptible to developing Th2 induced disease (Strachan, 2000). First, Strachan (1989) mentioned that the hypothesis was proposed to explain the observation that hay fever and eczema were less common in children from larger families, which were presumably exposed to more infectious agents through their siblings, than in children from small families, especially without siblings. Many bacteria and viruses derive a Th1-mediated immune response, which down-regulates Th2 responses. The urban-rural gradient in prevalence has been demonstrated most strongly in children who grew up in environments with a wide range of microbial exposures, who are protected from childhood asthma and atopy (the predisposition to develop IgE against common environmental allergens) in proportion to their level of exposure to bacterial and fungal microbes (Ege et al., 2011).

In association with the airway epithelium and underling mucosa is a specialized population of antigen-presenting cells (APCs) called DCs (Holgate, 2012). As allergen sensitization, DCs take up the allergens and present small peptide from them. DCs express receptors of the innate immune system and process allergens into small peptides and then present them through the major histocompatibility complexes, MHC class I and MHC class II for recognition by T cell receptors. In allergic individuals, it is promoted by interaction of the allergen with IgE attached to FcεRI, the high-affinity receptor for IgE (Sallmann et al., 2011). When individual is born, there is no DCs in the airway. Damage to and activation of the respiratory epithelium are the major stimuli that initiate the ingression of immature DCs from the bone marrow (McWilliam et al., 1994) and cause the release of C–C chemokines which direct DCs migration toward the epithelium and underlying mucosa (Hammad et al., 2010). GM-CSF, which is released from epithelial cells and immune cells in the presence of IL-4 and TNF-α, leads to DCs maturation to a fully competent as APCs. During initial allergen entering to airways to sensitize, Th2 lymphocyte differentiation from naїve T cells requires IL-4 release. The cellular source of the IL-4 is still unclear. There are some hypotheses to explain that (Holgate, 2012). Polarization to Th2 cells subtype is also under epigenetic regulation. From the study with mouse, microRNA-21 has been shown to exert a pivotal role in setting a balance between Th1 and Th2 responses. It works through binding the promoter of the gene encoding IL-12 p35 and inhibiting its activation in favor of a Th2 profile. Conversely, reduced microRNA levels lead DCs to produce more IL-12, and allergen-stimulated T cells to produce more interferon-γ (IFN-γ ) and less IL-4, enhancing Th1 delayed-type hypersensitivity (Lu et al., 2011).

### DENDRITIC CELL ACTIVATION

As described above, DCs present small peptide from antigens through MHC class I and II/ T cell receptors. Once sensitized, T cells drive the allergic response in progress through interactions with DCs (Veres et al., 2011). DCs spread their processes into the lumen between airway epithelial cells and can detect allergen by forming tight junctions, keeping the epithelial barrier (Blank et al., 2011). In mouse, two distinct DC subsets have been described in accordance with their expression of the CD11c as myeloid [conventional DCs (cDCs), CD11c^+^] or plasmacytoid DCs (pDCs, CD11c^-^; Lambrecht and Hammad, 2009). Similarly, human DCs are subdivided into CD11c^-^ pDCs and CD11c^+^ myeloid DCs. Induced sputum from asthmatic airways and peripheral blood contain increased numbers of both pDCs and cDCs, which further increase in number upon allergen challenge (Dua et al., 2010). Proteolytic activities of allergens initiate to mature DCs. In a few hours after contact with allergen, pattern-recognition receptors activation, such as Toll-like receptors (TLRs) on DCs augments their homing capacity by upregulating chemokine receptors. It is cDC subtypes that are predominantly responsible for antigen presentation. Mature DCs shape an immunological synapse with the allergen-specific T lymphocytes to initiate a Th response (Holgate, 2012). Whereas some of the Th cells make their way to the B-cell follicle to facilitate immunoglobulin class switching from IgM to IgE, others move back to the airway mucosa to elicit the classical Th2 response through the secretion of the proallergic cytokines. Pattern-recognition receptors have a crucial adjuvant role in directing allergen sensitization. TLRs are key components of the innate immune system that mediate recognition and response to pathogen-associated molecular patterns (PAMPs) in the form of microbial, fungal and viral products and their ligands, including endotoxin which is recognized by TLR4, lipoproteins (TLR2 and TLR6), viral double- and single-stranded RNA (TLR3 and TLR7/8) and bacterial CpG-containing DNA (TLR9) (Akira et al., 2006). Other pattern recognition receptors respond to endogenously generated damage-associated molecular pattern molecules (DAMPs) produced during tissue damage. Inflammatory DCs have been suggested to be necessary and sufficient for the development of Th2 immunity to house dust mite allergen when the first exposure occurs by inhalation. For inhaled allergens, it is proposed that DCs amplify the Th2 immunity through basophiles and, in part, influenced by innate signaling through TLR4 and C-type lectin signaling on epithelial cells and DCs (Trompette et al., 2009). A cooperation of airway epithelium and DCs controls asthma development Th2 activation requires DCs-mediated antigen-presentation. Then, allergic sensitization fails to develop in the absence of DCs (Hammad et al., 2010), while DCs remain inactive in the absence of TLR ligation (Perros et al., 2009). That is, TLRs activation on epithelial cells enhances DCs motility and antigen sampling through the production of Th2-promoting chemokines and cytokines (IL-25, IL-33, GM-CSF).

### VIRAL INFECTION TO PREDISPOSITION

The fact that early-in-life sensitization to multiple allergens carries the greatest risk for developing asthma (Simpson et al., 2010) brings the question of what factors result in a predisposition to this phenotype. Although infection with rhinovirus is the major cause of acute exacerbation, in those genetically at risk of asthma, rhinovirus-induced wheezing in the first three years in the life is also the greatest risk factor for developing asthma at 6 years of age (Jackson et al., 2008). Impaired TLR3-mediated IFN-β and -λ production by asthmatic epithelial cells would make susceptible to both viral infection and allergic sensitization (Wark et al., 2005; Contoli et al., 2006; Bosco et al., 2010; Jartti and Korppi, 2011). Reduced primary IFN production by lower-airway epithelial cells enables some viruses to replicate, leading to cytotoxic cell, release of inflammatory products and enhanced viral shedding. Such events provide a strong stimulus for recruitment of immature DCs and their priming for allergen sensitization (McWilliam et al., 1994, 1996). When asthmatic epithelial cells are received to damage by rhinovirus infection, the cells generate increased amounts of the pro-Th2 cytokine thymic stromal lympoietin (Uller et al., 2010), which stimulates DCs and increases allergic inflammation, whereas exogenous IFN-b applied to asthmatic epithelium exerts anti-Th2 as well as antiviral properties (Cakebread et al., 2011).

### CELLULAR IMMUNITY

**FIGURE 2:**
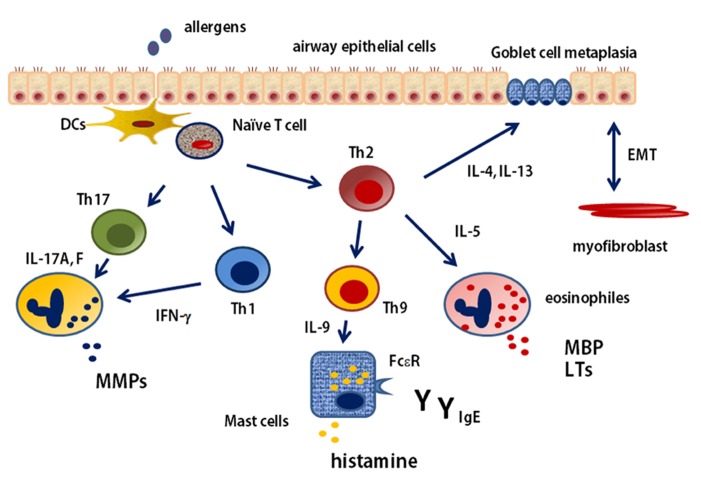
**T cell immune response in the asthmatic airways**. naїve T cell is received allergen presentation by DCs. The pathway begins with the development of Th2 cells and their production of the cytokines IL-4, IL-5, and IL-13. These cytokines stimulate allergic and eosinophilic inflammation as well as epithelial and smooth-muscle changes that contribute to asthma pathobiology. Th9 cell can be induced and stimulate mast cells by IL-9. naїve T cell is also differentiated to Th1 or Th17 cells depending on the existence of cytokines in the microenvironment. Th1 cell and Th17 cell stimulate and induce neutrophilic inflammation. EMT, epithelial-mesenchymal-myofibroblast transition; MMP, matrix metalloproteinase; MBP, major basic protein; LT, leukotriene.

Asthma is classically considered Th2 disease, with increased IgE and eosinophilic inflammation caused by increased levels of Th2-type cytokines. However, this paradigm has been challenged because of the realization that strategies designed to suppress Th2 function are not effective for all patients. The clinical phenotype of asthma is notoriously heterogeneous. It is shown that cellular immune process in the asthmatic airways in **Figure 2**. Th2 cells activation requires antigen-presentation by DCs. DCs play a role both in the initiation and maintenance of allergic airway inflammation and asthma, and control many aspects of the disease, including bronchial hyperresponsiveness and goblet cell metaplasia, by controlling the recruitment and activation of Th2 cells (Lambrecht and Hammad, 2009; Schuijs et al., 2013). Researches in both mouse and human, mentioned the expression of Th2-type cytokines, such as IL-4, IL-5, and IL-13, in the allergic lung. Experimental asthma models indicate that these cytokines, IL-13 in particular, are critical in driving key pathologic features of the allergic response. Moreover, Th2 blockade is very effective in suppressing these features of allergic disease in mice (Finkelman et al., 2010). The classical asthmatic phenotype is one of eosinophilia concomitant with high IgE levels. However, a proportion of patients are not atopic and do not have eosinophilic inflammation. In fact, it is estimated that as many as 50% of adult patients are encompassed by this non-atopic, non-eosinophilic, non-IgE-dependent subgroup (Lloyd and Saglani, 2013). Molecular therapy data support an overall Th2 association with phenotypes, such that they might satisfy a definition of Th2-associated asthma. However, even these distinctions are too simple, especially when disease severity is considered. Although children with severe asthma have eosinophilic inflammation, high-dose steroids effectively suppress Th2-type cytokines, such as IL-13 and IL-5, but symptoms remain with persistent eosinophilia (Bossley et al., 2012), thus raising the importance of identifying other less steroidsensitive, non-Th2 mediators driving disease. Then, it is apparent that asthma can no longer be considered simply a Th2-mediated disease.

**FIGURE 3:**
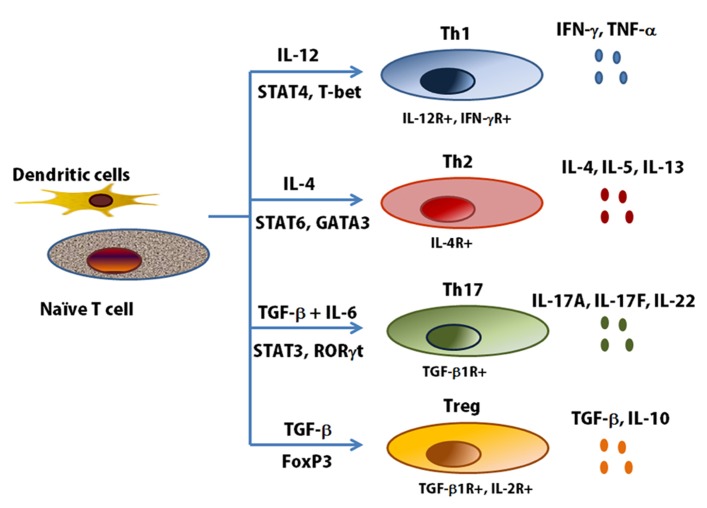
**T helper cell subsets and cytokine profiles**. Th1, Th2 and Th17 cells are a separate lineage of CD4+ T cells, distinct from other T cell subsets. Every specific T helper cells produce its specific cytokines (Lazarevic and Glimcher, 2011). T-bet, T-box expressed in T cells; FoxP3, forkhead box P3; ROR, retinoid-related orphan receptor.

Effector CD4 cells expressing IL-17A, IL-17F were first described in 2005 (Harrington et al., 2005; Park et al., 2005) and were thought to represent a distinct T-cell lineage that promoted the first revision of the Th1/Th2 paradigm of immunity. Differentiation of naive effector T cells in the presence of IL-6 and TGF-β, leading to the expression of the transcription factor RORγt, results in IL-17 expression through the transcription factors Smad 2/3, signal transducer and activation of transcription (STAT) 3, and nuclear factor κB. naїve T cells can differentiate several cell types and have specific immune response through the release of cell-type specific cytokines (**Figure 3**). Th17 cells have a role in regulating both neutrophilic and macrophage inflammation in autoimmune disease, and more recently they have been suggested to be involved in asthma and corticosteroid insensitivity (Nembrini et al., 2009). Conversely, their differentiation is restricted by both Th1 and Th2 cytokines including IFN-γ, IL-4, and IL-13 (Park et al., 2005). Specifically, the induction of CXCL8, a potent neutrophil chemokine whose expression is elevated in airway secretions in severe asthma, has directly implicated Th17 cells in neutrophilic airway inflammation. IL-17A itself, but not IL-17F or IL-22, enhances the contractile force of ASM. Sensitized mice lacking the integrin αvβ8 on DCs show reduced activation of this IL-17A-linked pathway with antigen challenge. This reduction in smooth muscle contraction in the airways is reversible by IL-17A, indicating involvement of this cytokine on allergen-induced AHR by acting directly on ASM (Kudo et al., 2012). Allergic induces a strong Th17 response in association with airway neutrophilia and hyperresponsiveness, and this response is abrogated in IL-17F knockout mice (Yang et al., 2008). However, although a good case can be made for IL-17A and IL-17F in mouse models of neutrophilic and corticosteroid-refractory lung responses to allergens, evidence for IL-17 involvement in human asthma is less robust, despite some emerging genetic evidence and a potential role for IL-17A and IL-17F in moderate-to-severe disease (Chakir et al., 2003; Doe et al., 2010). In humans, a subset of Th2 memory and effector cells has been recognized expressing both GATA3 and RORγ t and, as a consequence, producing both Th17 and Th2 cytokines (Cosmi et al., 2010). Studies have reported that the number of circulating Th17 cells as well as plasma concentrations of IL-17 and IL-22 increase in proportion to disease severity. In a bronchial biopsy in asthma vs. normal controls, there was no correlation between IL-17A or IL-17F expression and the extent of neutrophilia, nor any link to asthma severity (Doe et al., 2010). The contribution of Th17 cells in human asthma has not been established enough. It is required to clear association of Th17 cells and subphenotype in human asthma. 

## CYTOKINE TARGETS

### IL-4/IL-13

The key cytokines involved in Th2-type immunoreaction are those encoded in the IL-4 gene cluster on chromosome 5q31, containing the genes encoding IL-3, IL-4, IL-5, IL-9, IL-13, and GM-CSF (Bowen et al., 2008). The fact that the Th2 pathway is crucial to asthma pathophysiology has been the driving force for a range of biologics targeting the specific cytokines. The signals of Th2-cell-associated cytokines, IL-4 and IL-13, transmit through the IL-4Ra/IL-13Ra1 complex. IL-4 promotes B-cell isotype switching, the upregulation of adhesion molecules, eotaxin production, and the development of AHR and goblet cell metaplasia. In animal model, IL-4 deficient mice were shown to be protected from developing asthma (Brusselle et al., 1994). IL-13 can have most of these functions (Wills-Karp et al., 1998; Webb et al., 2000). Furthermore, those cytokines have the potential to induce TSLP, GM-CSF, and CCL20 production by the airway epithelium (Reibman et al., 2003; Kato et al., 2007). Furthermore, IL-13 was shown to have direct effect to enhance ASM, upregulating RhoA protein which stimulates Rho-kinase inducing calcium sensitivity (Chiba et al., 2009). Therefore, a good example is the IL-4 and IL-13 pathway for anti cytokine treatment against asthma.

Given the clear evidence for IL-4 and/or IL-13 in mouse models of disease were launched and a humanized anti-IL-4 neutralizing antibody (pascolizumab) was introduced and showed promising results in human-derived cell lines and monkeys (Hart et al., 2002). However, IL-4-specific antagonists used in clinical trials have failed (Wenzel et al., 2007). More recently, a human monoclonal anti-IL-4Ra antibody (AMG317) has been developed but did not show clinical efficacy (Corren et al., 2010). For IL-13, several neutralizing antibodies have been developed, but trials are still in their infancy. The latter IL-13-antibody (CAT-354) has recently been shown to be safe for use in humans in a phase I clinical trial but its real clinical efficacy remains to be proven (Singh et al., 2010). Attempts to validate importance of IL-13 in human asthma revealed that only 50% of individuals with asthma had elevated IL-13 levels in sputum, irrespective of the severity of the disease (Berry et al., 2004). And Woodruff et al. (2009) have also shown that only 50% of patients express IL-13-responsive genes in the airway epithelial cells, and this is linked to a strong Th2 response in bronchial biopsies, as opposed to in other asthmatics, whose IL-13-responsive gene expression was almost same level from that of normal subjects. Th2-high subjects had greater expression of IL-13 in bronchial biopsies along with greater AHR and higher serum IgE, blood and airway eosinophilia. It was suggested that one IL-13 biomarker was periostin (Woodruff et al., 2009). In a recently published trial, the monoclonal antibody (mAb) to IL-13, lebrikizumab, when administered to patients with chronic moderate-to-severe asthma for 12 weeks, significantly increased baseline spirometry (5.5%). This result was enhanced in those with elevated serum periostin (high periostin 8.2% vs. low periostin 1.2%; Corren et al., 2011).

### IL-5

IL-5 is a key cytokine crucial to eosinophil growth, maturation, activation, and survival whose blockade in various animal models has a strong effect on acute and more sustained pulmonary eosinophilia and attendant changes in lung function. It is mainly produced by Th2-lymphocytes, mast cells and eosinophils. Interestingly, IL-5 regulates its own receptor expression during eosinophil ontogeny consisting of an IL-5- specific receptor α-chain, and common β-chain. Because of its restriction to the eosinophil/basophil lineage in humans, IL-5 therapy may attenuate key characteristics of allergic airway inflammation, such as airway eosinophilia, airway remodeling, and AHR, without affecting the function of other immune cells (Trifilieff et al., 2001; Flood-Page et al., 2003; Humbles et al., 2004). It has also been implicated in the induction of AHR, as IL-5 inhalation by asthmatic patients induces eosinophil influx and AHR (Leckie et al., 2000). However, despite markedly reducing both circulating and sputum eosinophilia, two humanized mAbs, mepolizumab and reslizumab, when administered to patients with moderate-to-severe asthma, had no overall effect on any asthma outcome measures. Nonetheless, the studies of mepolizumab for patients with severe asthma requiring oral corticosteroids and persistent sputum eosinophilia showed a good clinical response (Haldar et al., 2009; Nair et al., 2009), as also found in Churg-Strauss and other hypereosinophilic syndromes (Abonia and Putnam, 2011). Similar results have also been obtained with reslizumab (Castro et al., 2011; Spergel et al., 2012). Efficacy of mepolizumab has also been described in severe eosinophilic nasal polyposis in proportion to nasal lavage IL-5 levels (Gevaert et al., 2006). A further development of this approach has been the introduction of a highly active mAb targeting IL-5Rα (benralizumab), which has been defucosylated to enhance its antibody-dependent cell-mediated cytotoxicity potential (Kolbeck et al., 2010). The studies demonstrate that anti-IL-5 therapy is effective in reducing exacerbation frequency in severe asthma, with highest efficacy in subgroups of patients where eosinophils have a pathogenic role. A phase 1 study in mild asthma has shown a strong dose-related reduction of circulating eosinophils lasting 8–12 weeks after a single injection (Busse et al., 2010). It seems, however, that for the majority of asthmatic patients the anti-IL-5 treatment will need to be administered in combination with other therapies that suppress asthma features through other mechanisms. Results of clinical trials targeting the IL5Rα subunit to obtain long-term depletion of eosinophils and basophils are eagerly awaited.

### IL-17/IL-22

The rapid emergence and characterization of the Th17 lineage (CD4 T cells producing IL-17 family; IL-17A, IL-17F, IL-22) refines the existing model and provides a more unified perspective of allergic inflammation by CD4+ T cell subsets. Interestingly, some asthmatic individuals, especially those poorly responding to steroid treatment, show airway infiltrations primarily composed of neutrophils. These cells are probably recruited to the airways by IL-17-producing cells that also produce IL-4 (Wang et al., 2010a). In mice, allergic sensitization followed by challenge of the airways induces a strong Th17 response and IL-17 controls bronchial hyperresponsiveness and airway remodeling, and some of these effects are mediated directly on bronchial smooth muscle cells (Pichavant et al., 2008; Wang et al., 2010b; Bellini et al., 2012; Kudo et al., 2012). Moreover, IL-17 can also induce steroid insensitivity in bronchial epithelial cells (Zijlstra et al., 2012). IL-22 can also be produced by Th17 cells. In mouse asthma models, IL-22 seems to exert a dual role. Indeed, IL-22 blockade in Th2 sensitization dramatically reduced eosinophil recruitment, Th2 cytokine and chemokine production, AHR, and mucus production. In contrast, IL-22 inhibition in allergen challenge induced lung inflammation and increased Th2 cytokine production. On epithelial cells, IL-22 has the potential to induce the production of antimicrobial peptides and to promote epithelial repair as well as suppressing the production of proinflammatory chemokines and cytokines (Pennino et al., 2012). Despite these studies, our knowledge of IL-22 in asthma pathophysiology is still limited.

IL-17A has been considered as one of most important player in asthma, however, clinical attempts for anti-IL-17A therapy to asthma has just begun (**Table 1**). Any data in anti-IL-17A trials for asthma are not available so far. Some clinical trials targeted at IL-17A have conducted and substantiated importance of IL-17A in autoimmune disorders. Phase II data on secukinumab, ixekizumab, and brodalumab in psoriasis indicate rapid and pronounced effects on measures of disease activity (Hueber et al., 2010). Early clinical trials in psoriatic arthritis, rheumatoid arthritis, and ankylosing spondylitis also support the therapeutic utility of IL-17A inhibition.

**Table 1:** Monoclonal antibodies against IL-17 pathway clinical trials.

mAbs	Description	Phase	Indications
Brodalumab	Full human IgG2/anti IL-17RA	II	Asthma, Ps, PsA, RA Secukinumab
Secukinumab	Full human IgG1K/ anti IL-17A	III	Ps, PsA, RA, AS
		II	veitis
Ixekizumab	Humanized, hinge-modified IgG4/anti IL-17A	III	Ps
		II	RA
		I	PsA
Ustekinumab	Full human IgG1/anti p40 of IL-12/23	III	Crohn’s, PsA
		II	AS, sarcoidosis, cirrhosis
		Approved	Ps
CNTO 1959	Full human mAb/anti p19 of IL-23	II	PsA
MK-3222	Humanized mAb/ anti p19 of IL-23	II	Ps
AMG 139	Full human mAb/ anti IL-23	I	Crohn’s, Ps
RG4934	Humanized mAb/ anti IL-17A	I	
NI 1401	Full human IgG1 mAb/ IL-17A/F	I	
SCH 900117	Humanized mAb/ IL-17A	I	

*Ps: psoriasis; PsA: psoriatic arthritis, RA: rheumatoid arthritis; AS: ankylosing; spondylitis.*

In addition, whereas secukinumab and ixekizumab selectively target and neutralize IL-17A, brodalumab binds the IL-17RA chain of the heteromeric IL-17 receptor, which is shared with multiple members of the IL-17 cytokine family and is therefore expected to inhibit the biological activity of IL-17A and IL-17F as well as IL-17C (Ramirez-Carrozzi et al., 2011), IL-17E (IL-25) and potentially other not yet discovered IL-17 family members that utilize IL-17RA (Papp et al., 2012). Considering with these data from clinical trials for autoimmune disease, this hypothetical advantage for IL-17A inhibitors against asthma can be expected to have clinical benefits. We have to wait that data from asthma studies becomes available.

### IL-9

Interleukin-9 produced from CD4+ T cell (Th9) has been identified as a subset definite from the classical Th2 cells, requiring the transcription factors IRF4, PU1, STAT6, Smad3, and Notch signaling for development. The cells differentiate in response to IL-4 and TGF-β and are described to promote T cell proliferation, IgE and IgG production by B-cells, survival and maturation of eosinophils, increasing the number of mast cell (Veldhoen et al., 2008; Staudt et al., 2010; Kearley et al., 2011; Elyaman et al., 2012; Goswami et al., 2012). Studies in human have also shown that IL-9 expression increased markedly in response to allergen challenge (Erpenbeck et al., 2003) and IL-9 is highly expressed and localized to tissue lymphocytes during intestinal parasite infection (Faulkner et al., 1998) and to CD3+ cells in bronchial submucosa and BAL (Shimbara et al., 2000). In studies using IL-9 transgenic and knockout mice, direct IL-9 instillation into the lungs and blocking mAbs, it has been shown that IL-9 drives mucus production, both by a direct effect on airway epithelia (Bryce, 2011) and also by interacting with IL-13 (Steenwinckel et al., 2007). Mice with IL-9 overexpression in lung have increased airway inflammation and AHR (Bisgaard et al., 2007; Gern, 2011). IL-9 is also made by ILC2s and boosts production of IL-5 and IL-13 (Rabinovitch et al., 2005). Along with IL-4 and stem cell factor, IL-9 is also a potent stimulus for mast-cell development (Kearley et al., 2011). As IL-9 has been implicated in both inflammatory and remodeling components in mouse models of allergic airway disease, it seems an attractive therapeutic target. Currently, clinical data on anti-IL-9 therapeutics are modest and larger clinical trials are eagerly awaited to conclude whether this form of therapy can be used in the treatment of asthma (Shalev et al., 2011). Two first-in-human, open-label dose-escalation trials of a monoclonal antibody against IL-9, MEDI-528, in normal subjects and subjects with mild asthma have been successfully completed, showing some evidence of efficacy (Parker et al., 2011).

### TNF-α

Tumor necrosis factor α, a multifunctional cytokine that exerts a variety of effects, such as growth promotion, apoptosis, angiogenesis, cytotoxicity, inflammation, and immunomodulation, has been implicated in several inflammatory conditions. This cytokine is not only produced predominantly by activated macrophages but also by other immune (lymphocytes, natural killer cells, mast cells) as well as stromal (endothelial cells, fibroblasts, microglial cells) cells and presents in increased concentrations in bronchoalveolar fluid from the airways of patients with asthma (Broide et al., 1992). Some studies mentioned a relationship between TNF-α and severity of asthma.

The rates of death and complications are high among patients with refractory asthma and account for a disproportionate amount of the health resource burden attributed to asthma (Serra-Batlles et al., 1998). The airway abnormality in severe asthma is different from that in more mild asthma in having a more heterogeneous pattern of inflammatory response (Wenzel et al., 1999), with greater involvement of neutrophilic inflammation and the distal lung (Berry et al., 2005) and increased airway remodeling (Busse et al., 1999). Interest in the role of TNF-α in refractory asthma has been increased by a study showing increased concentrations of TNF-α in BAL from patients with more severe asthma and by an uncontrolled study showing that treatment with the recombinant soluble TNF-α receptor etanercept markedly improved AHR in patients with refractory asthma (Howarth et al., 2005). On the other hand, targeting TNF-α in severe asthma with golimumab yields responders and non-responders (Wenzel et al., 2009). And administration with infliximab for severe asthma also does responders and non-responders (Taillé et al., 2013). Therefore, controlled studies have shown controversial results and the risk-benefit profile of TNF-blocking agents is still debated (Cox, 2009).

The studies suggest that anti-TNF-α agents might improve the condition of a subgroup of patients severe steroid-dependent asthma, who have life-threatening exacerbations and complications of long-term steroid therapy. In the studies, the identification of more neutrophilic asthma that is less dependent upon Th2 mechanisms and, as a consequence, less responsive to corticosteroids might help identify a responsive target subpopulation. Such patients have been shown to have high circulating TNF-α and CXCL-8 as biomarkers (Silvestri et al., 2006). A transcriptomic analysis applied to induced sputum has identified a unique signature with prominence of TNF-α and nuclear factor-κB pathways (Baines et al., 2010). This stratification of asthma into pathway-selective phenotypes is likely to be a key driver for future drug development, as is proving so successful for cancer treatments (Holgate, 2012).

## CONCLUSION

Bronchial asthma is a world-wide common disease and characterized by reversible airflow limitation, with non-specific AHR related to airway inflammation. Airway inflammation induces not only asthmatic symptoms which are the reversible airway obstruction and ASM contraction but also airway remodeling. Lately, the information for airway remodeling is increasing, the number of myofibroblasts increases in the understructure of epithelium, the proximity of the smooth muscle layer and the lamina reticularis. And it is more understood what EMT is. EMT can play a important role in airway remodeling. These epithelial and mesenchymal cells cause persistence of the inflammatory infiltrate and induce histological changes in the airway wall, increasing thickness of the basement membrane, collagen deposition and smooth muscle hypertrophy and hyperplasia. Subepithelial collagens cause thickening and increasing density of the basement membrane.

Classically, asthma is considered as Th2 disease, relating to increased IgE and eosinophilic inflammation in the airway. Recent results have shown that not only Th2, but Th17 and Th9 cells subset also contributed the disease, releasing their specific cytokines. These different cytokine give different biological effect. These can be targeted as an anti-cytokine treatment in asthma and some monoclonal antibodies against specific cytokines or their receptors are available. The results of those clinical trials have said that trials failed to control disease, despite it was clearly confirmed that those cytokines contributed the disease in animal model studies. It is required that more information for subphenotype of human asthma and its mechanism in more detail.

## Conflict of Interest Statement

The authors declare that the research was conducted in the absence of any commercial or financial relationships that could be construed as a potential conflict of interest.
